# Recent Progress in Obstetrics

**Published:** 1880-08-20

**Authors:** W. L. Richardson


### RECENT PROGRESS IN OBSTETRICS.

BY W. L. RICHARDSON, M.D.

*Intra-Uterine Vaccination.*—Following out the idea suggested by Prof. Bollinger (Munich) in his monograph published in Volkmann's series, Dr. A. E. Burkhardt (Deutsches Archiv fur klinische Medicin) gives the results of some experiments made in the obstetric wards in the hospital at Basel.

During the years 1877 and 1878 he revaccinated twenty-eight pregnant women. Owing to circumstances beyond his control, only eight of the children of these women were available for future experiment. Four children were then vaccinated, whose mothers had not been vaccinated during the pregnancy, and in every case perfect pustules were produced. With this same lymph, whose efficacy had thus been proven, he vaccinated the eight children of the mothers who had been vaccinated during pregnancy.

The results were as follows: The children of four women, whose revaccination during pregnancy had been perfectly successful, were found to be insusceptible of the vaccine lymph. The children of two of the women, whose revaccination during pregnancy had been only partially successful, were also found to be proof against vaccination. Of the two children whose mothers had been unsuccessfully revaccinated during pregnancy, one was vaccinated successfully, and the other failed.

These experiments of Dr. Burckhardt, although few in number, agree with the results obtained by Rickett, who inoculated about seven hundred ewes during the last few weeks of gestation. Their lambs were inoculated, when from five to six weeks old, with sheep-pox lymph, with no result, although at the same time thirty-six lambs, whose mothers had not been inoculated, were all successfully operated on and had true pustules.

*Antiseptic Obstetrics.*—Prof. Stadtfeldt, of Copenhagen, calls the attention of the profession to the great value of antiseptics in the treatment of obstetric cases. He says (Centralblatt fur Gynacologie), that since 1870 he has employed this method of treatment, and with the most gratifying results.

During the last five years, out of 5098 lying-in patients, only one in 116 has died of puerperal fever. This result is more favorable than that obtained in any other lying-in institution. He advises that the vagina be carefully washed out before the delivery, and that the moment the presenting part of the child shows itself at the vulva a carbolic vapor spray be thrown on the exposed parts. This should be continued until any lacerarions occurring during the delivery are united by sutures. The vagina should then be carefully washed out with carbolized water, as also the vulva and adjacent parts.

In all cases in which the hand or instruments have been introduced into the vagina, or where a portion of the membranes have been left behind, a three per cent. carbolized intra-uterine injection should be used after the delivery. He has never seen the slightest evil result follow an intra-uterine injection, although he has employed them in hundreds of cases.

Dr. Shucking advises that immediately after the delivery the vagina be wiped out with a tampon of cotton which has been dipped in a solution of carbolic acid (five per cent.). A sound is then carried up to the fundus uteri, having previously been surrounded by a piece of gauze soaked in the same solution. The uterus and vagina are then thoroughly disinfected by means of an irrigator, which is connected with the sound. For permanent irrigation he uses a solution containing ten per cent. of sulphite of soda and five per cent. of glycerine. Every twelve hours the sound and gauze are removed and a fresh instrument is inserted, while at the same time the carbolized injection, followed by the sulphite of soda wash, is repeated.

*Laceration of the Perinæum.*—Dr. T. A. Reamy, in Obstetric Gazette, calls the attention of the profession to the necessity of immediately sewing up a perinæum which has been torn during a delivery. The parts should be sponged with warm water, and if the flowing at all impedes the operation, a sponge can easily be inserted into the upper part of the vagina. There is no necessity for an anæsthetic, as the pain amounts to nothing; nor is there any for trimming the parts. The sutures should be placed very closely together, at least four to the inch. By placing them so closely, there is not the necessity of drawing the edges so tightly together. All clots and blood must be removed from between the parts before they are brought into apposition. The urine should be drawn for two or three days. It is not at all necessary to confine the patient's legs together, nor to insist upon her lying in one position; but she should not be allowed to separate them widely.

*Porro's Operation.*—Dr. R. J. Kinkead, in a recent paper read before the Dublin Obstetrical Society, gives an interesting and critical description of this operation as compared with Cæsarean section, laparo-elytrotomy, and craniotomy.

Thus far thirty cases have been reported, with the result of fourteen mothers being saved and sixteen deaths, or a mortality of 53.3 per cent. The dangers which attend Cæsarean section are :

1. Peritonitis. 2. Metritis. 3. Hæmorrhage. 4. Septicæmia. 5. Shock. 6. Intestinal obstruction, arising from a coil of the intestine being caught in the uterine wound.

By Porro's method, the second, third and sixth of these are abolished; the fourth is greatly reduced, and, with the proper use of antiseptics, should be entirely abolished. The danger from peritonitis is also greatly reduced. Several modifications of the details of the operation have been advised by various operators, but the main point consists in a combination of the Cæsarean section with amputation of the supra-vaginal portion of the uterus, at a level with the os internum.—*Boston Med. and Surg. Journal.*

